# Living with autism without knowing: receiving a diagnosis in later life

**DOI:** 10.1080/21642850.2019.1684920

**Published:** 2019-11-06

**Authors:** Steven D. Stagg, Hannah Belcher

**Affiliations:** Department of Psychology, Anglia Ruskin University, Cambridge, United Kingdom

**Keywords:** Autism spectrum disorders, diagnosis, older adults, late diagnosis

## Abstract

Increasingly adults over the age of 50 are receiving a diagnosis of autism spectrum condition. Growing up in a time when autism was poorly recognised, these adults have lived unknowingly with the condition and face readjustment. This paper reports the first study to investigate this population. Nine adults over the age of 50, who had recently been diagnosed with ASC, were interviewed, and thematic analysis was used to analyse the transcripts. Results showed that the participants had received treatment for anxiety and depression. They reported ASC behaviours in their childhood and growing up they felt isolated and alien. Receiving a diagnosis was seen as a positive step and allowed for a reconfiguration of self and an appreciation of individual needs. Given the positive aspects of receiving a late diagnosis, more work is needed to identify older adults with undiagnosed ASC.

Autism spectrum condition (ASC) affects individuals differently, but core behavioural features in social communication and restricted and repetitive behaviour are standard identifiers of the condition (American Psychiatric Association, 2013). The term autism spectrum disorder was first included in the third edition of the Diagnostic and Statistical Manual of Mental Disorders (DSM-III, APA, 1980), and the classification has been refined in subsequent editions. Since its inclusion in the DSM, the prevalence rate of ASC has been continually revised upwards (see Idring et al., 2015). Without a clear definition of the behavioural features of ASC existing in the past, individuals with ASC born before 1980 may have gone undiagnosed or misdiagnosed (Brugha et al., 2011; Geurts, Stek, & Comijs, 2015). This paper focuses on the experience of receiving a diagnosis of ASC in later life and is one of the first to document the experiences of adults over the age of 50.

**Figure 1.**
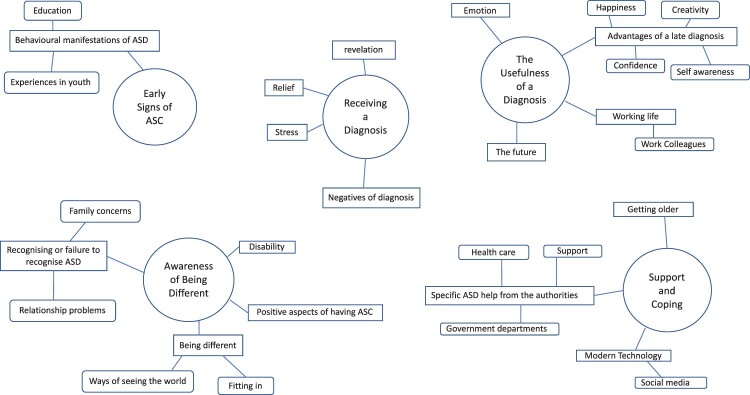
Final thematic map showing five main themes and corresponding first and second-order codes.

Research into undiagnosed older adults is essential as many individuals, who eventually go on to receive a diagnosis of ASC, are already being treated for social problems, anxiety and mood disturbances (Bishop-Fitzpatrick et al., 2018; Geurts & Jansen, 2012) without their core disorder being recognised. Mental health concerns are cited as a primary reason for adults eventually receiving a diagnosis (Jones, Goddard, Hill, Henry, & Crane, 2014). Additionally, adults with ASC face isolation and poor employment prospects (Howlin & Moss, 2012), and health and educational services for adults with ASC remain underdeveloped (Murphy et al., 2016) with 62% of adults with ASC reporting lack of support (Bancroft, Batten, Lambert, & Madders, 2012).

Receiving a diagnosis of ASC in later life will necessarily pose challenges to self-concept and possible futures (Morris, 1985). Taylor’s (1983) cognitive adaptation model suggests that individuals faced with life-changing information need to re-evaluate their sense of self and their possible futures. This re-evaluation involves three mechanisms: meaning-making, taking control and self-esteem building. The model suggests that individuals need to examine the causes of the life-changing event and consider how this change alters their current meaning system. They need to re-evaluate and gain control over their current circumstances and build self-esteem. Taylor suggests that comparing oneself to others undergoing the same situation is helpful in this respect. The experience of receiving a diagnosis can also be mediated through experience and self-awareness. If individuals are aware of the problem they have, then the diagnosis is less likely to be a source of shock, and in these cases, the primary experience of the process may be one of relief (Peel, Parry, Douglas, & Lawton, 2004).

Research into receiving a diagnosis in the context of ASC has traditionally used surveys and focused attention on how parents experience the diagnostic process (e.g. Soriano, Hill, & Crane, 2015). Few studies have sought to understand the experiences undergone by older adults. This gap is not surprising, given that many older adults are possibly living without knowledge of their condition. Qualitative research has tended to focus on younger adults ranging from teenagers to adults in their early forties; although diagnosis in these studies was often confirmed at a younger age (Crane et al., 2018; Jones, Zahl, & Huws, 2001; Molloy & Vasil, 2004; Punshon, Skirrow, & Murphy, 2009). This body of research has highlighted some key phenomena of the diagnosis process. For example, themes include the need to re-evaluate identity and produce new narratives to make sense of current and past experiences (Molloy & Vasil, 2004; Punshon et al., 2009); an enhancement of social esteem (Jones et al., 2001); and lack of post-diagnostic support (Crane et al., 2018).

To date, no study has investigated the phenomenon of receiving a diagnosis exclusively in middle and older middle age. Research into the experience of the diagnostic processes has tended to focus on third rather than first-person accounts (Connors & Stalker, 2007; Khine, 2003; Sloper, 2002) or has used a survey approach. Both approaches are valid, but surveys tend to frame responses, and they are often devised without the input of those taking part in the study. The survey approach risks missing important information. We wished to centre our research firmly on the lived experience of the individuals receiving the diagnosis and investigate how they experienced their diagnosis. In this respect, our first intention was to describe and document experience.

## Method

### Reflexive comment

The initial motivation for the research, driven by a lack of relevant studies, was to document the experiences of older adults with ASD. During the interviews, the issue of receiving a diagnosis emerged as a prominent concern. This particular element of their experience prompted the focus of the current paper. A fundamental approach to the research was not to see autism as a deficit, as a way of being less than, and to avoid viewing autism through psychological concepts such as cognition and development. Instead, we aimed to allow participants to show us what aspects of their lives and their psychological functioning were essential to them and their families. As a discipline, psychology has a tendency to thrust its growing concerns onto the subjects of study rather than first taking the time to describe and understand phenomena from the subject’s viewpoint. Both researchers had received different diagnoses of developmental disorders later on in life, and it was important that our personal experiences did not lead the interviewees or the analysis. For these reasons, the free-associative narrative approach (Hollway & Jefferson, 2008) was used during the interviews and thematic analysis used to analyse the transcripts.

## Design

Interviews were conducted using the free-associative narrative interview technique (Hollway & Jefferson, 2008). The narrative interview technique allows the interviewee to guide the direction of the interview, thus allowing the focus and agenda of the interview to change depending on the interviewees own experience. In this respect, we attempted to avoid imposing a narrative on the interviewee, which may occur through overly directive questioning (Mishler, 1991). Guiding questions were asked (see Appendix A), but participants had the freedom to take the interview in the direction they wished; our follow up questions focused mainly on following the narrative provided by the interviewee. The resulting transcripts were analysed using thematic analysis.

### Participants

Nine participants were recruited through online ASC forums and messages posted on a blog run by the second author, and recruitment took place over three months. Receiving a diagnosis in middle-age remains uncommon, and participants for the study were difficult to recruit. Participants had received a diagnosis from a trained clinician through the National Health Service in the U.K, and the majority has been diagnosed within two years of taking part in the study. One participant received a diagnosis ten years before the study and one had received a diagnosis seven years before the study. All participants were between 52 and 54 years of age, five of the participants were female, and four were male. Seven of the participants were married or in committed relationships, and two were currently single. Seven of the participants had one or more children, six of the participants were in full-time employment, two were unemployed, and one had retired. Pseudonyms have been assigned to each participant. When asked why they had sought out a diagnosis, three participants suggested it was because they had a child that had been diagnosed and they recognised similar symptoms in themselves; two of the participants had partners that had asked them to seek a diagnosis; two were referred for a diagnosis by a therapist who was treating them for a different condition; and two went for a diagnosis after a friend or colleague suggested that they may have autism. All participants received extensive information before the study, and informed consent was obtained in writing. The research received ethical approval from the Departmental Research Ethics panel at Anglia Ruskin University.

### Procedure

For some people with ASC social contact and face-to-face communication can be daunting and may provoke extreme anxiety (Bellini, 2004); therefore, participants were given the choice of how and where the interview was conducted. In this manner, the research could take accounts from individuals with ASC who might self-exclude when faced with traditional research and interviewing methods. One participant was interviewed by phone, one by Skype (with video-enabled), and one through an extended email exchange; the six remaining participants were interviewed face-to-face at places of their choosing. One interview took place in the participant’s home, one took place in a quiet area of a hotel lobby, and four took place in an office within the university. Both authors conducted all but three of the interviews; SS conducted two interviews, and HB conducted the email interview. The interviews lasted between 45 min and one hour. All interviews were audio-recorded for transcription.

### Data analysis

Interviews were transcribed, and coding was applied to the transcripts. The interview conducted by email was compiled into a single document. The analysis followed the approach set out by Braun and Clarke (2006) for conducting thematic analysis and followed their six phases of analysis. The analysis incorporated a critical realist framework, whereby semantic themes were developed. This approach acknowledges the experience of the participant and how meaning is derived from experience and the influence the social world has on this process (Braun & Clarke, 2006). The theoretical stance meant that the reports, experiences, meanings and reality of the participants were fully recognised.

Following a detailed reading approach (Van Manen, 1990), initial codes were generated using an inductive approach (Frith & Gleeson, 2004) by which the smallest units of meaning were extracted from either a few words, phrasal group or entire sentence rather than picking out phrases that directly related to receiving a diagnosis. This method effectively prevents the imposition of meaning onto the data at the early stages of coding. The coding was carried out using NVivo software and initially produced 49 first-order codes, which were grouped into second-order codes based on the similarity of meaning or experience. Codes not related to aspects of receiving a diagnosis were excluded. In the second stage of analysis, we began to group similar or overlapping codes, and when complete, these groupings were named as themes. Themes that emerged relating to general issues involving work (codes were related to general aspects of work) and neighbours (codes related to problems with noise from neighbours and not liking neighbours) and socialising (code related to general issues relating to problems socialising and forming friends) were not included in the analysis. These themes generally reflected issues concerning ASC rather than specific issues relating to receiving a diagnosis.

The analysis involved a recursive process, with second-order codes and themes checked against the participant’s accounts and adapted until a fit between the accounts and the themes was achieved. We then used these themes to inform further analysis of the data, looking for any missed information. See Figure 1 for an outline of the final thematic map.

## Results

Although participants did not necessarily employ a chronological order when speaking about their experiences, the themes have been loosely ordered around chronology. Five superordinate themes emerged from the transcripts. These were:

1. Early signs of ASC
2. Awareness of being different
3. Receiving a diagnosis
4. The usefulness of a diagnosis
5. Support and coping

### Early signs of ASC

As children, the participants were conscious of their differences, but they were unable to reflect on why their behaviour isolated them. Characteristic symptoms of ASC were readily present, including social isolation, repetitive behaviours, and fondness for routine.

I’ve never had friends; I’ve never made friends. (James)

I had difficulty with changes to routines, or unexpected situations. Even if the changes were minor, I found it difficult to deal with them. (Brenda)

The social thing was a problem, I couldn’t do birthday parties, and I couldn’t really behave with other kids for more than about 10 min. (Paul)

I was completely isolated and always in trouble, pretty strongly disliked by a lot of people … I tried to be friends with people and just got rejected. I don’t know why. (David)

My mum said when I was at infant school, I couldn’t cope with working in the classroom with other kids, and I used to have to work in the library quite a lot. (Linda)

But it’s funny, I’ve got cine-film of me in my bedroom, and you can see everything was in place. Even though all my clothes were clean they would all have to be washed and just so on the hangers. You can actually see me doing that on this film, putting everything in, and you can see where everything is ordered in my bedroom. (Debra)

### Awareness of being different

Studies have reported that individuals with ASC often seek a diagnosis due to social interaction and relationship concerns (Jones et al., 2014). One constant point expressed by participants in our study was the feeling of always having been different from others. The inability to ‘fit in’ was endured until it became untenable.

I’d always felt like this alien … . I feel like I’m a different type of human to non-autistic humans. (Brenda)

I just didn’t fit in, and I felt terrible. (James)

I always knew I was different, I always knew I didn’t fit in, but I didn’t realise what it was, the label that went with it. (Mary)

Knowledge of being different brings with it a search for explanations. Before receiving a diagnosis, some of the participants expressed their difference in terms of a negative. This negative labelling grew from a logical appraisal of the situation: ‘If so many people despise me, I must be a bad person’.

I just thought I was just bad really and didn’t really fit in and people didn’t like me, and I couldn’t really understand why. I suppose I thought I was different but wrong but didn’t understand what was wrong. (David)

I thought maybe I’m a bad person, I’ve got a horrible personality, there’s something about me people don’t like, and I didn’t understand why. (Brenda)

I was starting to get comfortable with the idea that I don’t fit in, but I didn’t think of Asperger’s I just thought I was naughty by nature. (Robert)

### Receiving a diagnosis

For the participants, the early reactions to receiving a diagnosis are a combination of different emotions. Initially, participants felt the diagnosis vindicated their feelings of being different and provided a reason for past experiences. The diagnosis allowed them to let go of impossible struggles and reframe their self-identity.

It really was like a sort of eureka moment … it was kind of a relief … and it wasn’t my fault, and that was one of the biggest things, that I realised it wasn’t my fault. (Brenda)

It’s the relief of knowing what’s wrong, or what has been wrong. (Linda)

A relief, because for years and years everything has been put down to anxiety and depression. Everything from the last 30 years made sense, it just all fitted in and it made sense. (Debra)

So it was a real (sighs) I mean ‘revelation’ is not even the word, really, it was (sighs) I was just stunned, really, to think that I could have gone through life potentially having Asperger’s and never having realised. (Mary)

Jones et al. (2001) suggested that the diagnosis may enhance an individual’s self-esteem, and this may be the case if the diagnosis is made early on in life. In our study, feelings of self-esteem were not mentioned, and the participants focused more on the challenge of coming to terms with a new lens through which to view the self.

Initially I was pleased. Then I think I was in shock for a while, and it was like having a complete reboot of my self-perception, which was a bit … I don’t know what I felt because I haven’t identified it … Probably a bit panicky, and then disappointed, really … I think I’m in a period now of still a bit of shock of the … not shock, but adjustment to what the diagnosis would mean. (Linda)

I went through several stages of feeling … First of all, I was thinking … It was strange, because although I knew it, I kind of felt some sort of disbelief as well. And there were times also, not long after as well, I felt angry and thinking why me? And other times it was the relief, and other times I was pleased. So it was a lot of different emotions, really. I think there’s always going to be an element of the why me, so it sort of robs you of that right to be like everyone else. (Brenda)

### Usefulness of the diagnosis

The diagnosis enabled the participants to place their behaviour within the perspective of an ASC framework. In some cases, the diagnosis provoked discoveries and new explorations of self, as the individual set about acknowledging and repairing certain perceived deficits. For David, the diagnosis allowed him to understand better his reaction to external stimuli, and while understanding an aversion to sunlight may seem obvious and trivial this was something that David perceived to be important and spoke about in some length.

Now I know that allegedly if you’re just, you don’t like bright light, allergic to it. I don’t know, it’s like I have explanations for things now, I never used to have. (David)

The greater level of self-awareness that comes with a diagnosis allows individuals to have more control over their lives and manage the way the react to situations.

Being aware of it [ASD] has enabled me to plan and prepare for situations, knowing how I may react, and how to avoid difficult situations … so I can keep to places and activities I am comfortable with. (Paul)

In the case of Robert, the diagnosis has unexpectedly helped him cope with his severe asthma attacks. The once nebulous causes for anxiety onset are now grounded in a narrative of ASC. This has allowed Robert to wean himself off the inhaler and has given him control over the illness.

Now because I know officially about the condition, I can say oh right I’m getting wheezy probably because I’m also getting anxious and now you know I could never go anywhere without my puffer in this pocket and my other puffer in the other pocket and whatever and now I think do I need to take it? (Robert)

For Linda, the diagnosis has explained her poor understanding of emotion. Knowledge of the problem has enabled her to put in place a strategy for learning about emotions.

Since my diagnosis it’s been fantastic. It’s a breakthrough for me now to … Somehow, it’s helped me start to tick off emotions. I’ve realised what these feelings are that I have … . I’ve identified a couple of months ago, guilt. For the first time I’ve realised what guilt feels like, and I can look back on my life and see all the times I’ve felt guilty but didn’t know … Yes. It was a revelation. Fantastic. (Linda)

Not all aspects of receiving a diagnosis have been positive for the participants. With the discovery of new elements of self also comes a realisation of the limitations the condition places on the individual.

It robs me, I guess, of maybe confidence and stuff. Maybe I would have, if I hadn’t got it, I would have maybe done some different things in my life. (Brenda)

It’s been really difficult to maintain a feeling of sense of worth. (Mary)

James perceived the diagnosis as resulting in the loss of his job through discrimination experienced from his employer.

She started bullying me quite seriously from then on, and within about eighteen months I was out of a job, and I think if I hadn’t bothered finding out what Asperger’s was, I would have just been this lonely person who just carried on. I sometimes wonder whether I should have, is it a bad thing to have had, the diagnosis. (James)

### Support and coping

In Britain, support for individuals with ASC is enshrined in ‘The Autism Act’ (UK Parliament, 2009) and the ‘Strategy for Adults with Autism’ (Department of Health, 2010). However, older adults are often overlooked by support mechanisms, and they tend to report receiving inadequate support (Bancroft et al., 2012). This lack of support was echoed by the participants. When asked about specific support Robert replied:

There’s none, there’s none you know there’s none that is positive you know it, the support that’s out there, except for voluntary groups and people that you meet on Twitter and so on, the support that’s out there is couched in this mental health you know, mental illness sort of frame work. (Robert)

I registered as disabled, but then … I don’t know what point that’s … They just sent me back a card. It didn’t even have my name on it. Maybe anyone can write in for a disabled card. (Linda)

I did have a consultant psychiatrist, and the one time when I was really bad, around Christmas time, I contacted him and he never got back to me. (Debra)

I am thinking this autistic spectrum condition might just be phased out soon because it’s too expensive to recognise, that’s what I am wondering. (Susan)

## Discussion

This paper highlights critical themes related to the experience of receiving a diagnosis of ASC in later life and the antecedents to that diagnosis. It is the first paper to report the alienation that older adults feel living without knowledge of their condition and the first to look at an older age group. The paper documents the unique experience of being faced with a life-changing diagnosis, which directly challenges the individual’s sense of self, their past narratives and their future possibilities. Given the historical context of the disorder, and the progress made in aetiology and the development of diagnostic techniques, it is probable that there will be many adults receiving an ASC diagnosis after the age of 50, and it is crucial that health care professionals, social workers and clinicians spot signs of ASC in older adults.

Our study highlights that critical indicators of ASC in older adults can be found in their experiences of childhood. These indicators need to be taken into consideration when care professionals deal with cases of anxiety and depression in older adults in order to rule out possible non-diagnosed ASC. It is common for these elements to be supplied by a child’s parents during a formal diagnosis (Falkmer, Anderson, Falkmer, & Horlin, 2013), but older adults with autism may not have living parents, and their recollections of early experiences should be considered. Huws and Jones (2011) speak of ASC as an ‘absent presence’ in the lives of people who go undiagnosed, and the participants in our study were acutely aware of being different as children and reported peer rejection and isolation. A key theme to emerge from our research, and not previously reported, was the feeling of being different to the extent of being an alien. This theme has not been reported in other studies focusing on receiving a diagnosis most likely because past research has focused on children. However, a feeling of being alien is common in ASC and is often represented on websites and blogs hosted by individuals with ASC (Jones et al., 2001). Our study suggests that isolation, and not belonging, along with obsessive behaviours, remain dominant memories in older adults and can be accessed directly from the client during a diagnostic interview.

While parental concerns about infants suspected of having an ASC often focus on language delay, sensory behaviour and motor development (Sacrey et al., 2015) these can be hidden in older adults who may have developed strategies to deal with primary ASD markers. We suggest that the present experience of individuals seeking a diagnosis needs to be carefully examined by health professionals and probed in greater depth. Most of our sample had jobs, one had created a successful company, and several had started families, and it was only through in-depth questioning that we were able to bring to the fore specific issues related to autism. Professionals focusing on stock autistic behaviours could easily miss these aspects. Given the heterogeneous nature of ASC, it is not surprising that some misconceptions about the condition remain in healthcare professionals (Heidgerken, Geffken, Modi, & Frakey, 2005). At first sight, the participants in our study did not adhere to traditional views of people with ASC. However, the feeling of being a stranger in a strange land had not dissipated with age. Older adults presenting with anxiety, depression and mental health issues along with failure to achieve in employment or lack of employment should be probed for signs of ASC. Sources of anxiety in adults with ASC may be different to sources in typically developing individuals, and health care professionals should look for anxiety related to changes in routines, the anticipation of change, and anxiety caused by sensory issues (Gillott & Standen, 2007).

Our research suggests that receiving a diagnosis should be followed up with professional help to enable the individual to manage the change to their life and the change to their identity. The ‘biographical disruption’ (Bury, 1982) experienced by the participants often led to a positive outcome, but this was not the case for all the participants. More support is needed to allow individuals receiving a late diagnosis to question their taken-for-granted assumptions and behaviours and to rethink their biography and self-concept. The re-evaluation of personal history is particularly necessary, given the negative past experiences all the participants recounted. While past research has also focused on poor aftercare (e.g. Crane et al., 2018), our study also highlights the needs for special consideration in the workplace. Work can be beneficial for people with ASC and can increase cognitive (García-Villamisar & Hughes, 2007) and social competencies (Belcher & Smith, 1994). Advocacy work, supported by The Equality Act (2010), can support this process by helping the newly diagnosed individual to reshape their work environment to best suit their needs, and our study allowed us a unique insight into how this may take place. For example, Robert can pursue his passion for social justice at work, knowing that his employer understands that his views can be less than nuanced and often uncomfortably forthright. Similarly, Linda is permitted breaks after meetings, which she finds exhausting, and takes respite in a local supermarket where she is soothed by the order and repetitive pattern of the products stocked on the shelves.

In line with Taylor’s model (1983), most of the participants entered a process of re-examining aspects of their past and redeveloping past narratives. A common theme across research into the diagnostic process is the lack of professional help available after the individual has received a diagnosis (Crane et al., 2018; Crane, Chester, Goddard, Henry, & Hill, 2016). None of the participants in our study had received help from trained therapists, and intervention at this stage could help make the re-evaluation process a more positive experience. Over the past 15 years, acts of parliament have been passed to support and safeguard individuals with ASC (UK Parliament, 2009; Department of Health, 2015). Although the current guidance and the previous guidance (Department of Health, 2010) encourage health authorities to provide individual assessments of support and care needs, the participants in our study did not feel supported. Jones et al. (2014) reported that many individuals with ASC faced hurdles both before and after their diagnosis was made. Health professionals showed a lack of understanding and held stereotyped views of ASC. The participants in our study found support from health and local authorities inadequate. The British government has also recognised the limited support individuals with ASC can face (Department of Health, 2010, p. 7), and the National Autistic Society (Bancroft et al., 2012) reports that only 28% of adults with ASC received useful information about where to go for help.

A novel aspect of the study was the highlighting of the role the internet and online support groups can play in the diagnostic process. When help was received, the participants reported that it often came from within the ASC community and through online ASC support groups. Feeling that you are not alone and that others understand your experiences is empowering and can be a significant part of building self-esteem (Taylor, 1983). Hurlbutt and Chalmers (2002) highlighted how adults with ASC reported feeling proud to be part of a unique culture. For the adults in our study, gaining a diagnosis later in life meant that they had only experienced being on the outside of a culture they failed to grasp, and they were unaware that an alternative and supportive culture of individuals with ASC was available. Receiving a diagnosis opened up this alternative culture to them and provided information and support. Employing individuals with ASC in the care and health system and utilising online communities may be an effective way of supporting those who gain a diagnosis later in life, and this remains an unexplored research area.

### Limitations

The current study is based on a relatively small sample size, and we acknowledge this creates issues around saturation of insight. Recruiting participants for interviews rather than surveys is time-intensive, and more participants could result in further themes emerging around the subject of being diagnosed later in life. Defining a sample size in qualitative research is problematic with limited agreement on numbers with samples ranging from 2 to 400 (Fugard & Potts, 2015) and themes emerging with as few as six participants (Guest, Bunce, & Johnson, 2006). We also acknowledge that the different methods of interviewing in this study (face-to-face, phone, skype, email) meant that participants were not all receiving the same experience; although they were all responding to the same questions. We believe that flexibility is necessary for ASC research and that the participant should ultimately choose the method of interview. In this way, we can gain information from individuals who may refuse to take part in a face-to-face study.

### Implications

The study reported in this paper suggests that receiving a diagnosis in later life can be a positive and beneficial experience. Clinicians and health workers need to be more aware of the possible signs of undiagnosed autism, to avoid misdiagnosing depression, anxiety or other mental health conditions. A thorough study of the client’s early childhood and their current relations (family, work colleagues) can help. More work also needs to be done to support older adults after they receive a diagnosis. They and their families need to be supported through this transitional phase, and emphasis needs to be placed on future potential and possibilities. It is not known how many older individuals with ASC remain undiagnosed, but given the positive nature of receiving a diagnosis, more needs to be done to identify these individuals. Future research needs to gain an estimate of the number of undiagnosed cases of autism in older adults, and this may partly be achieved by screening older adults who are currently accessing mental health services. In order to do so, current screening tools for autism may need to be adapted to cover current employment and relationship issues, childhood factors along with questions about common autism traits.

## Appendix A

Primary questions

When did you receive a diagnosis?

When did you first become aware you had autism?

Tell me how managing your autism has changed as you have grown older.

How has the diagnosis affected your life?

How does your autism impact on your daily life?

Tell me about the positive aspects of having autism?

Are there negative aspects?

Tell me about your future hopes and fears

Do you currently have a job? Can you tell me about your work?
